# 340. Infections in The Unhoused: A Descriptive Review of Unhoused People Presenting to The Emergency Room with Fever

**DOI:** 10.1093/ofid/ofad500.411

**Published:** 2023-11-27

**Authors:** Jessica M McFarland, Ruchi Rawal, Yueqi Yan, Geetha Sivasubramanian

**Affiliations:** UCSF- Fresno, Clovis, California; UCSF-Fresno, fresno, California; UC Merced, merced, California; UCSF Fresno, Fresno, California

## Abstract

**Background:**

Homelessness has been increasing in several cities in California. The US Department of Housing and Urban Development and the Fresno Madera Point in Time Homeless Count, estimates that 4,216 people were experiencing homelessness during the year 2022 alone in Fresno County (Fig 1). People who are homeless often lack adequate health insurance, access to medical care, and have low healthcare literacy. They are subjected to poor sanitation and overcrowded living conditions putting them at risk for communicable diseases. Comprehensive studies of febrile illnesses in unhoused patients are sparse. We retrospectively studied unhoused individuals who presented to our center with fever. We aimed to quantify and understand their underlying presenting infections.

**Figure:**
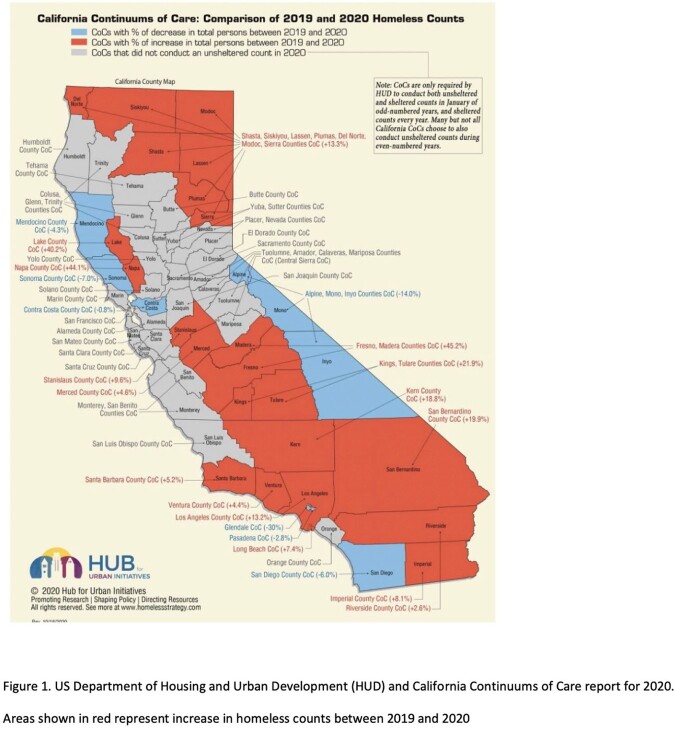

Areas shown in red represent increase in homeless counts between 2019 and 2020

**Methods:**

The Fresno Information Gateway (FIG) is an IRB (Institutional Review Board) approved database which has data captured on patients seen in our center. We used ICD 10 codes for unhoused, fever and subset of infections to obtain data between 2015-2019 (Fig 2). Crosstabulation and chi-square or Fisher’s exact test were performed to examine the frequency and percentage of individuals with each type of disease. T-test was used to compare the average age between samples with vs. without each disease. Logistic regression was performed to examine the association of factors with each disease, while controlling for other factors.

**Figure:**
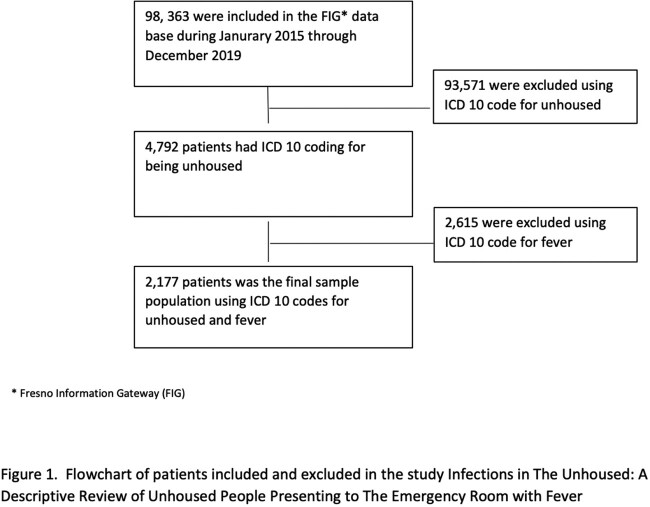

**Results:**

A total of 4792 unhoused patients were seen in the study period. Of these, 2177 patients presented with a fever (Table 1). Unhoused females had higher odds of presenting with blood stream and urinary tract infections (Table 2). Substance abusers had higher odds of soft tissue, cardiac and respiratory infections. Unhoused Asians or Pacific Islanders had higher odds of coccidioidomycosis, compared with their White counterparts.

**Figure:**
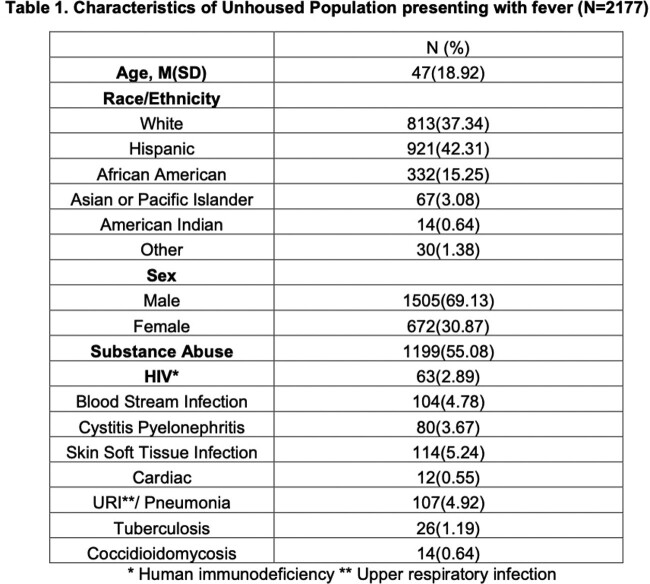

**Figure:**
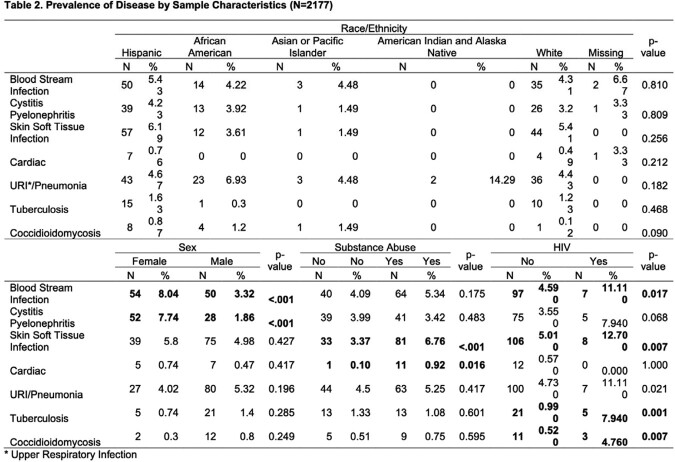

**Conclusion:**

A large number of unhoused patients presented to our center in Central California with febrile illness due to various infections. Unhoused females, substance users and HIV positive individuals in our study-set had higher odds of presenting with certain infections. Further studies are warranted to examine these associations, which may help us develop disease specific healthcare delivery models in unhoused patients.

**Disclosures:**

**All Authors**: No reported disclosures

